# YoMiCom framework for guild-based design of resilient microbial consortia in multi-stress agricultural systems

**DOI:** 10.3389/fpls.2026.1854447

**Published:** 2026-07-08

**Authors:** Abhishek Sharma

**Affiliations:** Amity Food and Agriculture Foundation, Amity University, Noida, India

**Keywords:** functional guilds, microbial consortia, plant-microbe interactions, sustainable agriculture, Yogic Microbiome, rhizosphere

## Abstract

Microbial consortia show promise for sustainable agriculture, yet their field performance often remains inconsistent due to ecological imbalance and instability in complex soil environments. The Yogic Microbiome (YoMiCom) framework addresses this challenge by presenting a systems-based approach to microbial consortium design, where plant-beneficial functions are organized into coordinated functional guilds that support ecological balance, compatibility, and resilience. By combining guild-based assembly with quantitative design metrics, such as the Functional Guild Index for strain prioritization and the Guild Balance Coefficient for functional distribution, within an iterative Design–Build–Test–Learn framework, YoMiCom shifts the focus from empirical assembly to structured, context-driven design. This framework is presented as a testable conceptual design approach that can be evaluated and refined across diverse agroecological contexts, thereby supporting the development of ecologically compatible and functionally balanced solutions for sustainable agriculture.

## 1 Introduction

Synthetic microbial community (SynComs) design has emerged as a powerful approach for understanding and constructing microbial consortia through omics-informed selection and controlled assembly of multiple strains (Armanhi et al., 2016; Vorholt et al., 2017). These approaches enable the identification of functional traits, interspecies interactions, and community-level behaviors under defined conditions, and have significantly advanced microbiome research. At the same time, they have contributed to a broader shift toward function-oriented thinking in microbial community design, in which the focus extends beyond individual strains to encompass coordinated functional roles within a consortium (Castrillo et al., 2017; Banerjee et al., 2019).

Although SynCom’s design is now conceptually well established, a key challenge lies in translating these advances into the development of microbial consortia that remain stable, adaptive, and effective under field conditions. Modern agriculture confronts escalating challenges from climate change, soil degradation, and intensifying pest pressures (Trivedi et al., 2017; Dastogeer et al., 2020), where microbial performance must be sustained across variable environments. In this context, the next critical step is not only to construct microbial communities but also to develop frameworks to design climate-resilient, functionally balanced consortia capable of consistent, predictable performance in real-world agricultural systems (Wang et al., 2025). Addressing this need, we propose the Yogic Microbiome (YoMiCom) framework, a systems-based approach for designing resilient microbial consortia through coordinated functional roles and ecological compatibility. The term draws upon the Yogic concept of union (yoga), denoting the integration of diverse yet interdependent elements into a regulated and balanced whole. Applied here, “yogic” designates a deliberately designed microbial ecosystem in which complementary functional members interact cooperatively and self-regulate without the dominance of any single taxon, thereby promoting resilience, adaptability, and coordinated community function. We employ the term as a design-oriented descriptor rather than a purely philosophical one: whereas conventional ecological terms such as “stability” or “diversity” characterize emergent states of an already-assembled community, “yogic” specifies an *a priori* design objective, the active pursuit of ecological harmony through harmonized functional complementarity and self-regulation, that guides consortium construction. This meaning is intended to remain interpretable to an international scientific readership independent of prior familiarity with the term. This manuscript is submitted as a Hypothesis and Theory article and presents a conceptual and theoretical framework rather than empirical data. All propositions are formally structured as falsifiable predictions in the **Supplementary Note**.

## 2 Defining the YoMiCom concept

Contemporary microbial consortium design in agriculture reflects a notable convergence toward function-oriented assembly yet exhibits divergence in how these functions are operationalized. Diverse approaches, including substrate-centric functional matching (Clagnan et al., 2023), bottom-up pathway engineering (Kurt et al., 2023), enrichment-based rhizosphere simplification (Chen et al., 2024), and host-mimic culturomics (Elsawey et al., 2022), independently emphasized the importance of functional organization in consortium design. Together, these developments indicate that while the field has converged on function as the basis for microbial consortium design, important challenges remain in translating these functions into structured design decisions. In most existing approaches, trait selection and community assembly are guided by empirical reasoning or omics-informed insights, but lack explicit quantitative frameworks to prioritize functional traits, allocate them across multiple stress conditions, and balance their representation within a consortium. As a result, critical design aspects such as functional trade-offs, dominance effects, and proportional contribution of different microbial groups are not systematically controlled during the assembly phase. Furthermore, although iterative Design–Build–Test–Learn (DBTL) cycles are increasingly adopted, the initial design stage often lacks measurable criteria to guide consortium construction in a predictive manner. These limitations highlight the need for a framework that introduces quantitative structure into the design phase itself, enabling more reproducible, balanced, and context-specific development of microbial consortia. YoMiCom builds upon existing practices of omics-informed strain selection and community assembly, but introduces an explicit design layer that prioritizes, organizes, and balances functional traits in a context-dependent manner. Central to this framework are the Functional Guild Index (FGI) and Guild Balance Coefficient (GBC), which enable the translation of trait-level information into measurable criteria for strain selection and community structuring prior to experimental validation. By integrating functional guild architecture with iterative Design–Build–Test–Learn (DBTL) cycles, YoMiCom shifts consortium development from descriptive, *post hoc* evaluation to structured, reproducible, and application-oriented design. The framework emphasizes ecological compatibility, functional redundancy, and adaptive resilience, thereby supporting the development of microbial consortia capable of consistent performance under multi-stress and variable field conditions. YoMiCom is an ecologically harmonious microbial consortium composed primarily of native plant-associated microorganisms, tailored to specific crops, soils, and climates. The consortium aims to enhance plant performance and stress tolerance through balanced, cooperative interactions among microbial members. Key processes include improved nutrient uptake, modulation of plant growth and stress responses, priming of plant defenses, increased tolerance to abiotic stress, and suppression of soil-borne pathogens. Together, these functions help sustain crop performance under variable environmental conditions (Kumar et al., 2022; Orozco-Mosqueda et al., 2023; Goszcz et al., 2025; Maake and Sibisi, 2025).

**Figure 1:**
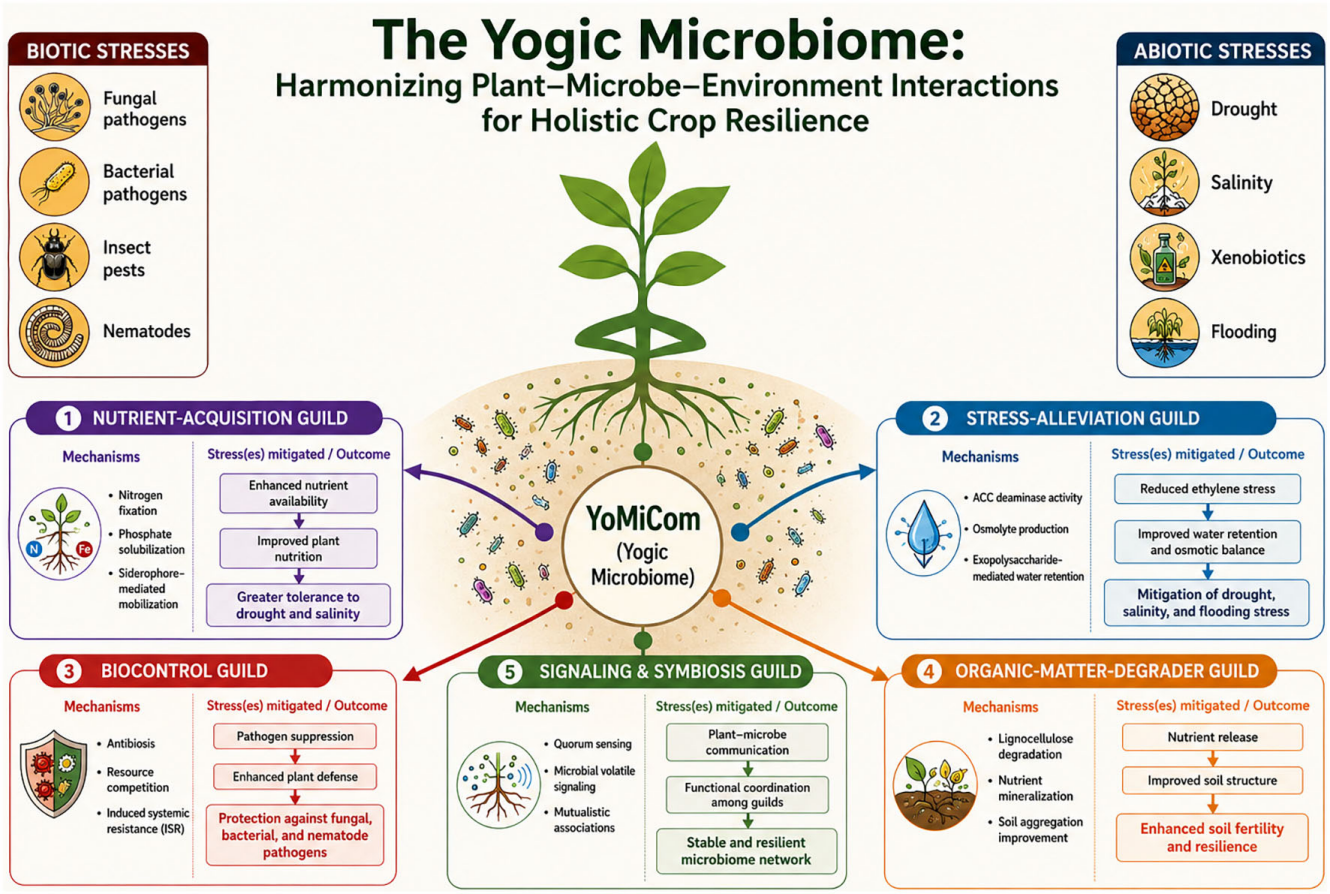
Yogic microbiome. This conceptual model shows a plant reaching a balanced state by aligning its interactions with the environment. The “Yogic Microbiome” in the root zone employs a suite of balancing mechanisms to counteract both biotic (e.g., fungi, bacteria, insects, nematodes) and abiotic (e.g., drought, salinity, xenobiotics, flooding) stresses, thereby enhancing overall crop resilience. The five functional guilds map onto specific stress-response mechanisms as follows: the nutrient-acquisition guild counters nutrient limitation through nitrogen fixation, phosphate solubilization, and siderophore-mediated iron mobilization; the stress-alleviation guild mitigates drought, salinity, and temperature stress through osmolyte production, ACC deaminase activity, and exopolysaccharide-mediated water retention; the biocontrol guild suppresses fungal, bacterial, and nematode pathogens through antibiosis, niche competition, and induced systemic resistance; the organic-matter-degrader guild improves soil structure and nutrient release through lignocellulolytic decomposition; and the signaling and symbiosis guild coordinates these functions through quorum sensing, volatile signaling, and establishment of mutualistic associations. (Original figure, created by the author for this manuscript.).

The foundation of the Yogic Microbiome rests on the yogic principle of balance through coordinated integration, in which stability arises not from the dominance of any single part but from the regulated interactions among them. In microbiological terms, this means the coordinated interactions among diverse microbial members that collectively promote stability and resilience under various biotic and abiotic stresses in the field (**Figure 1**).

## 3 The YomiCom framework

### 3.1 Core principles

YoMiCom operates on three foundational principles:

- Synergistic functionality: YoMiCom is based on the idea that microbes work best when they function together rather than alone. By combining microbes with complementary roles, microbial consortia can deliver stronger and more consistent benefits to plants than individual strains, as shown in studies on nutrient uptake and drought tolerance (Dnkha and Fattah, 2023; Sharma et al., 2023).
- Adaptive resilience: The framework emphasizes stability at the community level, allowing microbial consortia to maintain functional capacity under environmental perturbations through functional redundancy within guilds and phenotypic plasticity of constituent strains, as demonstrated in drought-stressed wheat systems (Tanveer et al., 2024).
- Plant–microbe–environment harmony: YoMiCom also prioritizes ecological compatibility with the surrounding soil ecosystem. By favoring native or well-adapted microbes, the framework supports plant stress tolerance while minimizing disruption to existing microbial communities (Schmitz et al., 2022).

### 3.2 Functional guild architecture

YoMiCom organizes microbial strains into five functional guilds, each addressing specific plant-microbe interaction domains:

a. Nutrient acquisition guild.

The members of this guild include nitrogen-fixing bacteria (*Rhizobium*, *Azospirillum*), phosphate-solubilizing microbes (*Bacillus*, *Pseudomonas*), arbuscular mycorrhizal fungi, and siderophore-producing bacteria, which collectively enhance the bioavailability of nitrogen, phosphorus, and iron, thereby improving plant nutrition and reducing fertilizer dependence (Faller et al., 2024; Lei et al., 2025; Rakhmatova et al., 2026).

b. Stress–alleviation guild.

This guild includes microbes that boost plant tolerance to drought, salinity, and extreme temperatures by producing osmolytes, stabilizing membranes, modulating phytohormones (e.g., reducing ethylene via ACC deaminase), or enhancing soil water retention through EPS production. Recent reviews and studies show drought- and salt-adapted rhizosphere microbes improve plant water status and yield under stress (Eswaran et al., 2024; De Silva et al., 2025).

c. Biocontrol guild.

Antibiotic- and lipopeptide-producing bacteria (e.g., *Bacillus*, *Pseudomonas*, and *Streptomyces*) and antagonistic fungi (e.g., *Trichoderma*) suppress pathogens through antibiosis, competition for niches and nutrients, parasitism, and induction of plant systemic resistance. Recent meta-analyses and lab–field translation studies have shown that well-assembled biocontrol guilds perform more reliably than single-strain products across various soils and climates (Raaijmakers et al., 2002; Roca et al., 2024; Zholdasbek et al., 2026).

d. Organic-matter-degrader guild.

Cellulolytic and ligninolytic bacteria and fungi collaborate to decompose crop residues, converting complex carbon compounds into simpler forms and releasing nutrients. This process improves soil structure and fertility. Advances in enzyme and metagenomic research have identified microorganisms and enzyme systems, such as cellulases, laccases, peroxidases, and cellulosomes, that enhance residue decomposition and support soil carbon cycling (Ding et al., 2025; Hsin et al., 2025).

e. Signaling & symbiosis guild.

Members facilitate communication among organisms through quorum sensing, volatile signals, and small molecules, thereby forming mutualistic symbioses with arbuscular and ectomycorrhizal fungi. They coordinate behaviors such as biofilm formation and priming, enhancing nutrient exchange and defense signaling between plants and microbiota. Recent reviews highlight plant receptors and signaling pathways that help plants distinguish and attract beneficial groups (Srivastava et al., 2024; Zheng et al., 2025).

The choice of these five guilds reflects their consistent empirical contribution to plant health across various crops and stress conditions. Recent meta-analyses show that these functions together account for over 80–85% of microbiome-mediated gains in yield, stress tolerance, and disease resistance reported under field conditions (Compant et al., 2019; Sessitsch et al., 2019; Francioli et al., 2025; Liu et al., 2025). Other processes, such as detoxification and carbon cycling, are regarded as subfunctions embedded within the stress-alleviation or degradation guilds. The guild framework is modular and can be expanded to include additional categories as crop- or environment-specific evidence develops.

## 4 Operationalization of the YoMiCom framework

**Figure 2:**
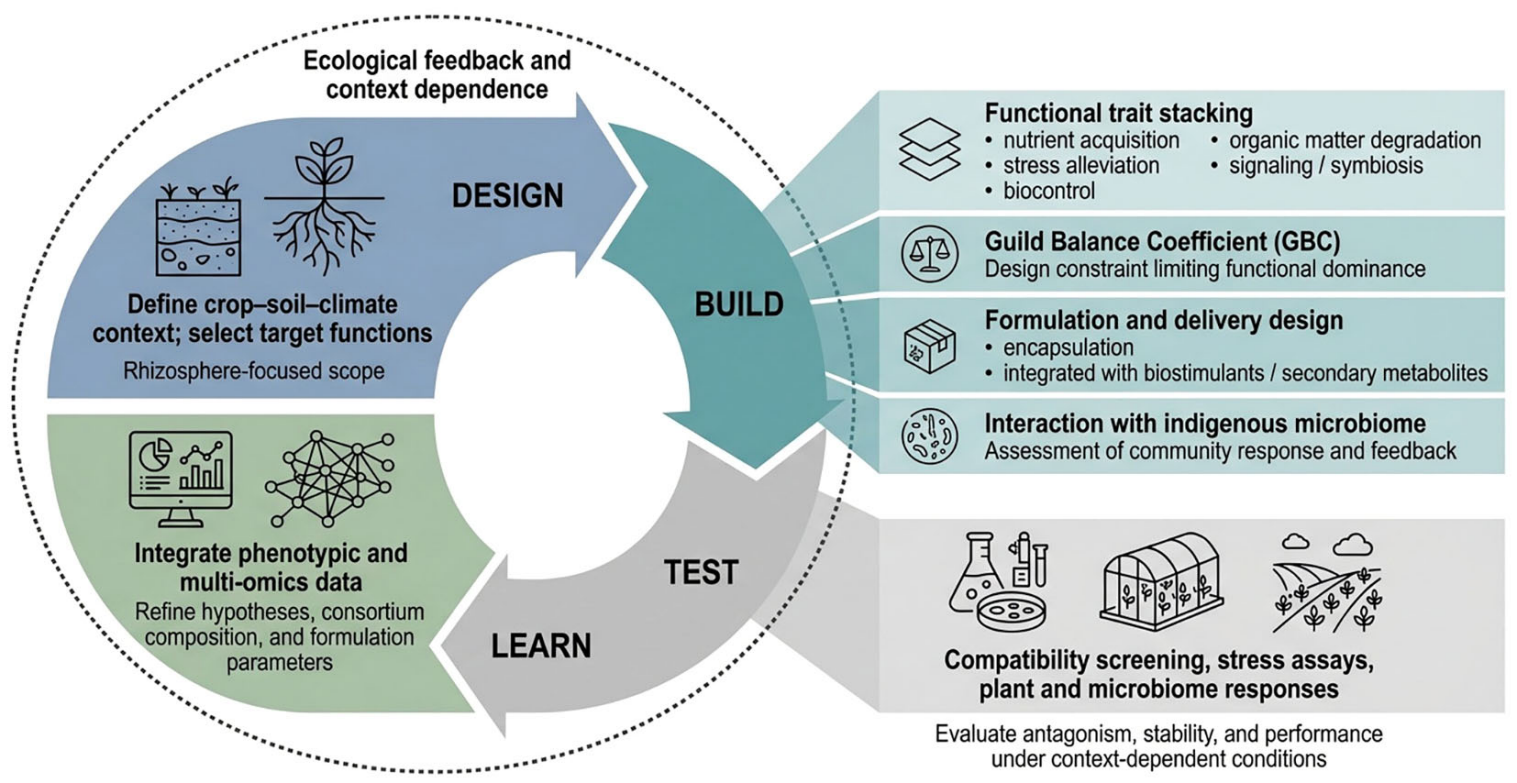
Yogic Microbiome (YoMiCom) formulation framework based on an iterative Design–Build–Test–Learn (DBTL) cycle. The outer dotted loop emphasizes ecological feedback and context dependence, highlighting the iterative and non-deterministic nature of the framework. (Original figure, created by the author for this manuscript.).

The idea of a “one-size-fits-all” microbial product is biologically and agronomically unrealistic. Because of the diversity of crops, soil chemistries, climate zones, and stress conditions, effective microbiome-based solutions need to be tailored to specific contexts. One organized approach to such customization is the Design–Build–Test–Learn (DBTL) cycle, adapted here as the Yogic Microbiome Formulation Framework (**Figure 2**).

The design phase begins by defining the target application (e.g., drought tolerance in maize or salinity tolerance in coastal rice). Indigenous microbial resources are identified through bioprospecting regionally adapted crops, focusing on the rhizosphere using established protocols (Toju et al., 2018; Compant et al., 2019). Candidate strains are isolated, then characterized using trait-based assays, molecular markers, and omics approaches. Metagenomics and metabolomics identify complementary traits, such as stress tolerance, pathogen suppression, nutrient mobilization, and phytohormone signaling, which inform the initial consortium blueprint (Deter and Lu, 2022). The build phase unfolds in five interlinked layers:

1. Functional Trait Stacking – The Build phase progresses through five interconnected layers, starting with functional trait stacking. Consortium assembly identifies distinct microbial guilds whose combined activities support complementary, plant-beneficial processes: nutrient acquisition, stress alleviation, biocontrol, organic matter degradation, and signaling/symbiosis. Each guild is defined by measurable traits (e.g., phosphate solubilization, ACC deaminase activity, antifungal activity, biofilm formation) relevant to improving crop resilience in specific agroecological settings (Jasso-Arreola et al., 2025; Lei et al., 2025). The framework prioritizes balanced functional coverage and compatibility among guild members while avoiding excessive redundancy that could cause competitive exclusion.

To operationalize guild-based consortium assembly, the Functional Guild Index (FGI) is introduced as a comparative metric for strain prioritization within a defined crop–stress context. FGI summarizes the weighted functional contribution of an individual strain within a specific guild based on trait performance:


$$FGI_{ig}=\sum_{t=1}^{T_{g}}{\text{w}}_{\text{gt}}\;{\text{T}}_{i,t}^{norm}$$

where *i* denotes an individual microbial strain, *g* denotes the functional guild, *t* denotes a measurable functional trait relevant to that guild, and *T_g_* is the total number of traits considered for the guild *g*. Here, ${\text{T}}_{i,t}^{norm}$ represents the normalized trait score of the strain *i* for trait *t*, and *w_gt_* is the context-dependent weight assigned to the trait *t* within guild *g*, with $\sum_{t=1}^{T_{g}}{\text{w}}_{\text{gt}}=1$. Normalization places all traits on a common 0–1 scale before aggregation, enabling comparison across diverse measurements.

Trait scores are normalized using min-max scaling, where each raw trait value is rescaled relative to the observed minimum and maximum across all candidate strains: Score_norm = (Score_raw − Score_min)/(Score_max − Score_min). This procedure ensures that no single trait dominates the FGI by virtue of its measurement scale rather than its biological relevance. To address the context-specificity of trait expression, trait weights (w_i) in the FGI formula are not fixed but are adjusted based on the prevailing abiotic stress profile of the target site. For example, in drought-prone systems, osmotic adjustment and root architecture traits receive greater weight than nutrient acquisition traits. Calibration of weights can be guided by published functional response data, regional soil characterization, or expert elicitation when site-specific empirical data are unavailable. The measurement protocols, assay methods, and gene markers used to quantify each trait are detailed in **Supplementary Table 1**.

FGI is calculated separately for each functional guild using only the trait set relevant to that guild. A single strain may contribute to multiple guilds depending on their functional profile; in such cases, FGI is computed independently for each guild. For example, *Trichoderma* may contribute to the biocontrol guild (antagonistic activity), stress-alleviation guild (induced systemic resistance), and organic matter degradation guild (cellulolytic enzymes), yielding separate FGI values for each domain. FGI serves as a comparative tool for strain selection rather than an absolute predictor of field performance. For instance, within the biocontrol guild, *Trichoderma* showing normalized trait scores of 0.8 (antifungal activity) and 0.7 (siderophore production) would receive a higher FGI than strains with lower scores, supporting its prioritization. However, final consortium composition requires balanced representation across guilds, as quantified by the Guild Balance Coefficient (GBC):

Before FGI scoring, candidate strains are screened for compatibility with the target host using available co-occurrence data, published host-range profiles, or documented rhizosphere competence. Strains with documented incompatibility, such as obligate symbionts applied to non-host plants, are excluded prior to scoring. This host-compatibility pre-screening step ensures that FGI rankings reflect strains that are biologically deployable in the target system, independent of their *in vitro* trait performance.


$$GBC_{g}=\frac{F_{g}}{\sum_{k=1}^{m}F_{k}}$$

Where *F_g_* is the aggregate functional contribution of the guild *g* (sum or mean of FGIs of assigned strains), *m* is the total number of guilds and *F_k_* is the aggregate functional contribution of each guild as the sum moves from the first guild to the *m*-th guild. By construction, the guild fractions sum to unity:


$$\sum_{k=1}^{m}GBC_{k}=1$$

Even GBC distributions across guilds indicate functional balance, whereas disproportionate values signal skewness that may compromise field performance, for example, when nutrient acquisition dominates without adequate stress alleviation or biocontrol. This rationale rests on the principle that multi-stress agricultural environments impose concurrent abiotic and biotic pressures, and that no single functional guild can simultaneously address nutrient limitation, pathogen suppression, and stress alleviation. Balanced functional coverage across guilds, therefore, provides broader adaptive capacity than functional depth within a single guild.

It is acknowledged, however, that the GBC-derived preference for functional balance does not apply universally. Certain agroecological applications are better served by consortia with a dominant functional specialization. Disease-suppressive soils, for instance, are often characterized by communities enriched in strains with highly specialized functions, such as antimicrobial compound-producing *Pseudomonas* or *Streptomyces*, in which functional skewness is the operative mechanism of efficacy. The YoMiCom framework accommodates this by allowing practitioners to adjust guild weights in the GBC formula based on the primary design objective. Where pathogen suppression is the dominant goal, the biocontrol guild weight may be intentionally elevated, shifting the GBC optimum toward a targeted rather than balanced configuration. The framework thus supports context-adjusted functional prioritization rather than imposing a single optimality criterion.

GBC functions as a comparative metric rather than a universal threshold, guiding iterative refinement during design. Trait weights *w_gt_* are derived from literature-based normalized evidence, such as relative yield loss, physiological indices, or enzyme activity under specific stresses, together with site-specific indicators such as rainfall, salinity, or pathogen incidence. In multi-stress scenarios, such as concurrent drought and salinity, initial weights can be assigned based on normalized stress contributions; for example, if drought and salinity contribute approximately 40% and 25% of relative yield loss, respectively, then w_drought_ = 0.615 and w_salinity_ = 0.385. These weights can then be refined through DBTL cycles as experimental evidence accumulates.

A simplified illustration of how FGI and GBC guide consortium selection is provided in **Box 1**. This worked example is framed within a representative multi-stress agricultural scenario to demonstrate how the metrics translate into a practical, crop-contextualized design decision.

Box 1 **Illustration of consortium selection using FGI and GBC**

Two candidate consortia (A and B), each comprising three microbial strains, are evaluated for deployment in a representative multi-stress agricultural scenario—for example, maize grown under combined drought stress and moderate soil-borne pathogen pressure—based on their functional distribution across three guilds: nutrient acquisition, stress alleviation, and biocontrol. The table below presents representative FGI values derived from normalized trait scores (0–1 scale), where higher values indicate stronger functional contribution within a guild.

**Table:** Table B1 | Representative FGI values for two candidate microbial consortia (A and B) across three functional guilds under a combined drought and soil-borne pathogen stress scenario in maize.

Guild	Consortium A	Consortium B
Nutrient acquisition	0.30	0.45
Stress alleviation	0.50	0.50
Biocontrol	0.70	0.55

The total functional contribution (the sum of FGI values across guilds) is equal for both consortia (A = 1.50; B = 1.50), but their functional distributions differ. The Guild Balance Coefficient (GBC) is calculated as the proportional contribution of each guild to the total functional capacity:

For Consortium A:

GBC ^(A)^ = [0.30/1.50, 0.50/1.50, 0.70/1.50] = [0.20, 0.33, 0.47]

For Consortium B:

GBC ^(B)^ = [0.45/1.50, 0.50/1.50, 0.55/1.50] = [0.30, 0.33, 0.37]

Consortium A shows a functional skew toward biocontrol, whereas Consortium B exhibits a more even distribution across all guilds. Accordingly, the YoMiCom framework prioritizes Consortium B because it achieves a better functional balance aligned with multi-stress field requirements.

Note: This example is illustrative and demonstrates how FGI and GBC serve as decision-support metrics to guide consortium design, emphasizing balanced guild representation rather than maximizing a single guild. All numerical values in this box are hypothetical and are used solely for illustrative purposes. They do not represent measured experimental data.

The FGI formula, as presented, employs a linear, additive aggregation of weighted trait scores. This is an intentional simplification appropriate for a pre-experimental design filter, where the goal is to prioritize relative strain over mechanistic prediction of community outcomes. A linear approximation provides computational tractability and interpretability at the strain-screening stage, before co-culture interaction data are available. It is recognized that microbial interactions within an assembled consortium are inherently non-linear, involving reciprocal facilitation, competitive exclusion, metabolic cross-feeding, and context-dependent antagonism that cannot be captured by summing independent trait scores. These non-linear dynamics are not neglected but are instead addressed empirically through iterative DBTL cycles: the Build and Test phases directly measure emergent consortium behavior, and the Learn phase updates strain selection and weighting based on observed interaction outcomes. Extensions of the FGI to incorporate consumer-resource models or pairwise interaction terms from generalized Lotka-Volterra frameworks are identified as priorities for the next generation of the YoMiCom computational pipeline.

2. Context-Specific Rhizosphere Consortium Assembly– Plants actively shape rhizosphere microbiota through root exudation, immune signaling, and host genetics (Compant et al., 2019). Under stress, selective enrichment of beneficial microbial populations enhances plant resilience (Ali et al., 2023), providing a biological basis for rhizosphere-targeted interventions.

The YoMiCom framework employs rhizosphere-centered, context-specific assembly of microbial consortia through three structured steps: (a) stacking functional traits, (b) estimating guild-level contribution using the Guild Balance Coefficient (GBC), and (c) assessing compatibility.

Compatibility is treated as a screening criterion, initially guided by co-occurrence patterns or trait overlaps and subsequently tested using co-culture or inhibition assays. In YoMiCom, harmonization is operationally defined as the absence of dominant functional imbalance and the preservation of complementary functional capacity at the community level. The framework does not seek to eliminate antagonistic interactions, which are intrinsic to microbial communities, but instead constrains their system-level impact through functional balancing and redundancy. By limiting the overrepresentation of any single guild, GBC reduces the likelihood that competitive dominance undermines consortium stability. Antagonism is thus positioned as an evaluative factor within iterative DBTL cycles rather than as a fully predictable design variable.

Strains carrying multiple high-scoring traits may be subject to metabolic trade-offs arising from the resource costs of maintaining broad functional repertoires. In ecological theory, generalists bear higher metabolic drag than specialists, potentially limiting their per-trait efficiency. The YoMiCom framework addresses this through the Build and Test phases of the DBTL cycle, in which *in vitro* and in planta performance data reveal whether high-FGI generalist strains deliver the predicted functional outputs. Where metabolic drag is empirically detected, the framework supports substitution of specialist strains with narrower but more efficient functional profiles, consistent with consumer-resource theory.

Community stability and resilience to perturbation are not solely functions of functional balance but are also governed by network topology and the presence of keystone taxa. Hub taxa that mediate disproportionate ecological interactions can stabilize community dynamics even when their individual FGI scores are not maximal. Consortium establishment is further interpreted through community assembly theory, in which the relative balance of deterministic selection, dispersal, ecological drift, and diversification governs which introduced and indigenous taxa persist, and through successional dynamics that progressively reshape community composition following inoculation. Network-based stability descriptors—including connectance, modularity, and the ratio of negative to positive co-occurrence links—are accordingly incorporated as quantitative readouts of community robustness during the Test and Learn phases. The YoMiCom framework treats network-level properties as emergent outcomes evaluated during the Test phase, using co-occurrence analysis and community profiling to identify keystone candidates for inclusion or protection across DBTL cycles. Simulation of community trajectories under environmental perturbations, using frameworks such as generalized Lotka-Volterra models or agent-based models, is identified as a future analytical priority to generate quantitative predictions that can guide decisions in the Learn phase.

Controlled-Release Delivery – Controlled-release delivery systems, including layered encapsulation and stimuli-responsive polymers, enhance microbial persistence and enable gradual establishment under varying environmental conditions. These systems respond to physicochemical cues such as temperature, pH, and enzymatic activity, affecting microbial availability in soil (Shen et al., 2023).

Within the YoMiCom framework, controlled-release technologies are enabling tools that enhance microbial protection and persistence. Field-oriented innovations suggest that improved formulation strategies may contribute to yield gains and reduced synthetic inputs, though outcomes remain context dependent (Beula Isabel et al., 2024; Pehlivan et al., 2025).

3. Co-encapsulation with Microbial Metabolites – Co-encapsulation strategies combine microbial metabolites or biostimulant components with live inoculants to promote early plant responses and establish microbial populations. Formulations can include osmolytes, lipopeptides, phytohormone-related compounds, or signaling molecules. Biostimulants, such as humic substances and seaweed extracts, promote plant growth and nutrient uptake (Lau et al., 2025), whereas additives such as lipochitooligosaccharides and nanomaterials enhance microbial adhesion and performance (Compant et al., 2019).

The YoMiCom framework addresses technical challenges, including microbial viability, component compatibility, release kinetics, and scalability, through a modular design, GRAS-certified biocompatible materials, and aqueous fabrication methods. Economic feasibility, scalability, and regulatory factors are incorporated as design constraints within DBTL cycles.

4. Interaction with Indigenous Microbiome – A key aspect of YoMiCom is how introduced consortia interact with native soil microbial communities. YoMiCom treats native microbiome responses as empirical outcomes assessed within the DBTL cycle. Prebiotic carriers and formulation components may affect indigenous microbial activity, potentially impacting nutrient cycling, carbon turnover, and stress-related functions (Alahmad et al., 2023), though effects vary with soil type, crop species, and environmental conditions.

Assessment involves comparing microbiome profiles before and after YoMiCom application using α-diversity metrics (Shannon or Simpson indices), β-diversity analyses (Bray–Curtis dissimilarity or ordination), and functional annotation (KEGG, COG, MetaCyc) to assess metabolic pathway changes. Outcomes indicating minimal displacement and retained functional redundancy suggest compatible interactions, while significant disruptions signal the need for iterative reformulation in subsequent DBTL cycles. Indigenous microbiome dynamics are thus incorporated as a feedback constraint.

The Test phase assesses candidate YoMiCom formulations through laboratory assays, controlled-environment experiments, and limited field evaluations. Assessments combine microbial- and plant-level results, including stress tolerance, antagonism screening, and agronomic metrics like yield, water-use efficiency, nutrient uptake, and pathogen suppression. Microbiome responses are tracked via community profiling to analyze stability and compositional changes.

In the Learn phase, phenotypic outcomes and multi-omics data identify taxa, traits, or configurations linked to performance. Insights inform iterative refinement of consortium composition, dose, carrier type, and release characteristics, with a focus on efficacy, feasibility, and scalability. Progress through DBTL cycles is viewed as evidence-based refinement rather than a straightforward path to deployment. The YoMiCom framework is structured around hypothesis-driven, falsifiable predictions linking consortium composition to measurable outcomes at the microbial, plant, and ecosystem levels. Detailed examples of testable hypotheses and associated quantitative parameters are provided in the **Supplementary Note**.

Machine learning approaches represent a high-priority extension of the YoMiCom Learn phase. Predictive models trained on trait-performance datasets from prior DBTL cycles can identify the most impactful consortia formulations for subsequent validation rounds, accelerating convergence toward optimal consortium configurations and reducing the number of empirical trials required. This integration is structurally compatible with the framework’s iterative architecture: outputs from Test-phase community profiling serve as training data, and model predictions inform Build-phase strain selection in the next cycle.

To position the YoMiCom framework within the landscape of microbial inoculant strategies, **Table 1** summarizes key differences in design philosophy across single-strain systems, empirical and omics-informed consortia, and the proposed framework. Existing approaches largely rely on trait identification and community assembly, with limited quantitative guidance for functional prioritization and balancing. The YoMiCom framework addresses this gap by introducing measurable design parameters (FGI and GBC) that structure functional allocation and support the development of stable, context-specific microbial consortia.

**Table 1:** Conceptual comparison of microbial inoculant design approaches, highlighting the transition from strain selection to quantitative community design.

Criterion	Single-strain inoculants	Current empirical consortia design	YoMiCom framework
Functional scope	Single dominant function	Multiple functions via combining strains	Structured multi-functionality via defined (expandable) guild architecture
Design basis	Trait-centric selection of individual strains	Empirical and/or omics-informed selection with limited quantitative optimization	Quantitative, context-driven design using FGI, GBC, and DBTL optimization
Multi-stress capacity	Restricted to the intrinsic functional range of a single strain	Possible if selected strains collectively address stresses, but not explicitly optimized	Explicitly designed through context-weighted trait prioritization and guild balancing
Formulation integration	Considered after strain selection	Considered primarily as a delivery step with limited linkage to functional design	Incorporated as a design variable to align microbial survival, establishment, and functional performance
Omics integration	Used for strain characterization	Used for strain discovery and community assembly	Used for strain discovery, with additional quantitative filtering and balancing (FGI–GBC)
Field translation	Often inconsistent under field conditions	Variable performance across environments	Designed for ecological compatibility, redundancy, and iterative validation

## 5 Conclusion: a call for systems thinking in microbial consortium design

Agricultural microbiomes hold significant potential for sustainable intensification, yet translating microbiome knowledge into reliable field applications remains a major challenge. While advances in omics-driven discovery and microbial community assembly have improved our ability to identify and integrate beneficial microorganisms, the design of stable, resilient consortia for field conditions remains limited.

The Yogic Microbiome (YoMiCom) framework is proposed as a systems-oriented design approach that builds on these advances by introducing a quantitative structure to organize functional traits within microbial communities. Through guild-based architecture, context-dependent trait prioritization, and balancing via the Guild Balance Coefficient within a DBTL workflow, YoMiCom provides a structured pathway for developing ecologically compatible and multi-stress-resilient consortia.

The YoMiCom framework is proposed as a testable and adaptable design concept for guiding microbial consortium development across diverse agroecological contexts. Its advancement will depend on systematic field validation, integration with regulatory and deployment pathways, benchmarking against existing approaches, and the development of accessible decision-support tools to support broader adoption of quantitative consortium design. Future work will prioritize simulation-based testing of community trajectories under environmental perturbations, integration of machine learning for consortia formulation prediction, and development of the FGI–GBC computational pipeline as an open-access decision-support tool.

## Data Availability

The original contributions presented in the study are included in the article/**Supplementary Material**. Further inquiries can be directed to the corresponding author.
