# Supplementary material for: YoMiCom framework for guild-based design of resilient microbial consortia in multi-stress agricultural systems
Source: Front Plant Sci. 2026 Jul 8;17:1854447. doi: 10.3389/fpls.2026.1854447 (PMC13388798; doi:10.3389/fpls.2026.1854447)
Supplement: Supplementary file 1 [file Table1.docx]

**Hypothesis-Driven and Quantifiable Structure of the YoMiCom Framework**

To ensure experimental testability, the YoMiCom framework links each DBTL cycle to clear, falsifiable hypotheses grounded in measurable microbial, plant, and ecosystem-level parameters. For a specific target function, the design phase develops hypotheses in the form:

*If a YoMiCom assembled with defined functional guilds is applied under specified conditions, the resulting outcome will differ from that of an appropriate control.*

Illustrative examples include:

- **Nutrient acquisition hypothesis:** Applying a YoMiCom enriched with high-FGI phosphate-solubilizing strains increases soil Olsen-P levels and plant phosphorus uptake compared to uninoculated controls.
- **Stress alleviation hypothesis:** A YoMiCom designed for drought-prone conditions enhances plant physiological measures, such as relative water content or chlorophyll fluorescence, under standardized stress treatments.
- **Microbiome compatibility hypothesis:** The introduction of a YoMiCom does not lead to significant decreases in native microbial α-diversity or functional redundancy compared to untreated soils.

These hypotheses are assessed using quantitative parameters integrated into the framework, including FGI and GBC metrics; qPCR-based quantification of functional guilds; metagenomic or functional gene profiling; enzymatic activities; plant physiological measurements; and yield-related traits. By explicitly linking consortium composition to measurable outcomes, YoMiCom functions as a hypothesis-driven framework that supports systematic testing, comparison, and iterative refinement, rather than as a prescriptive or guaranteed-outcome system.
